# Pupillary responses to short-wavelength light are preserved in aging

**DOI:** 10.1038/srep43832

**Published:** 2017-03-07

**Authors:** A. V. Rukmini, Dan Milea, Tin Aung, Joshua J. Gooley

**Affiliations:** 1 Center for Cognitive Neuroscience, Duke-NUS Medical School, 169857, Singapore; 2 Program in Neuroscience and Behavioral Disorders, Duke-NUS Medical School, 169857, Singapore; 3 Singapore Eye Research Institute, Singapore National Eye Center, 168751, Singapore; 4 Department of Ophthalmology, Yong Loo Lin School of Medicine, National University of Singapore, 119228, Singapore; 5 Department of Physiology, Yong Loo Lin School of Medicine, National University of Singapore, 117597, Singapore

## Abstract

With aging, less blue light reaches the retina due to gradual yellowing of the lens. This could result in reduced activation of blue light-sensitive melanopsin-containing retinal ganglion cells, which mediate non-visual light responses (e.g., the pupillary light reflex, melatonin suppression, and circadian resetting). Herein, we tested the hypothesis that older individuals show greater impairment of pupillary responses to blue light relative to red light. Dose-response curves for pupillary constriction to 469-nm blue light and 631-nm red light were compared between young normal adults aged 21–30 years (*n* = 60) and older adults aged ≥50 years (normal, *n* = 54; mild cataract, *n* = 107; severe cataract, *n* = 18). Irrespective of wavelength, pupillary responses were reduced in older individuals and further attenuated by severe, but not mild, cataract. The reduction in pupillary responses was comparable in response to blue light and red light, suggesting that lens yellowing did not selectively reduce melanopsin-dependent light responses. Compensatory mechanisms likely occur in aging that ensure relative constancy of pupillary responses to blue light despite changes in lens transmission.

Aging is associated with yellowing of the lens and reduced transmission of short-wavelength light. This arises from age-dependent accumulation of pigments in the crystalline lens that preferentially absorb blue light^1^. The accumulation of lens pigments and insoluble crystalline aggregates can lead to further discoloration of the lens, increased light-scatter, and progressive loss of lens transparency, eventually leading to the development of cataract and severe visual loss^2^. It is widely believed that attenuation of short-wavelength light by the aging lens can lead to diminished non-visual light responses (e.g., pupillary light responses, melatonin suppression, and circadian entrainment)^3^. This is because non-visual light responses are mediated by intrinsically-photosensitive retinal ganglion cells (ipRGCs) that contain the short-wavelength sensitive photopigment melanopsin (λ_max_ = ~480 nm)^4^,^5^. It has been estimated that the amount of 480-nm light that can pass through the lens in late adulthood (80-year-old lens) is less than a third relative to childhood^6^. This age-dependent reduction in short-wavelength light reaching the retina has been hypothesized to contribute to reduced circadian responses to light and sleep disturbances^3^,^7^,^8^,^9^.

Surprisingly, it has yet to be clearly demonstrated that non-visual light responses show selective deficits to blue light in nonpathological aging or in eyes with cataract. The aim of this study was to address this gap in knowledge, by comparing dose-response curves for pupillary constriction to blue light (469 nm) versus red light (631 nm) in young healthy adults and in older adults either with or without cataract. We hypothesized that aging and increased cataract severity would be associated with a greater reduction in sensitivity of the pupillary light reflex to blue light relative to red light, hence revealing a functional consequence of age-dependent yellowing of the lens for melanopsin-dependent photoreception.

## Methods

### Subjects

#### Young adults

A group of 68 subjects between the ages of 21 and 30 years, with normal or corrected-to-normal vision (corrective lenses between 6 diopters and −6 diopters) were recruited from the general population using online advertisements and flyers. Subjects were recruited if they reported no ophthalmic history. Eligibility was determined using a health screening questionnaire. Exclusionary criteria included use of medications that might affect the size or reaction of the pupil; presence of a medical condition or disease that might alter pupil size or the ability of the pupil to constrict; or history of disease affecting the iris, pupil, or optic nerve that would lead to impaired pupillary light responses. Subjects who had prior intraocular surgery (except laser surgery for correction of refractive errors) were excluded from the study.

#### Older adults

A group of 300 subjects aged ≥50 years were recruited from a community polyclinic and underwent a comprehensive ophthalmic examination, as described previously^10^,^11^. The grade of cataract was assessed using a slit-lamp microscope and classified using the Lens Opacities Classification System III (LOCS III)^12^. By comparing images to LOCS III reference standards for cataract severity, subjects were classified as having no cataract (grade 1), mild cataract (grade 2) or severe cataract (grade 3). The study was approved by the SingHealth Centralized Institutional Review Board, and all participants provided written informed consent. Research procedures adhered to ethical principles outlined in the Declaration of Helsinki.

### Chromatic pupillometry

All participants underwent a chromatic pupillometry test, as described in our previous work^10^. In short, the direct pupillary light reflex was measured by exposing the left eye to light and recording pupil diameter of the same eye (i.e., the ipsilateral eye). The right eye (i.e., the contralateral eye) was covered with an eye patch to prevent a consensual pupillary light reflex in the left eye. In light exposure trials that occurred consecutively, the left eye was exposed to either 469-nm (blue) light or 631-nm (red) light using a Ganzfeld dome equipped with LEDs as the light source. During the procedure, subjects were seated with their head position fixed by a chinrest. Each light exposure sequence was 4 min, and consisted of 1 min of darkness, followed by 2 min of light exposure, and then 1 min of darkness. During the 2-min light exposure period, the irradiance measured at the surface of the eye was increased gradually (from 6.8–13.8 log photons cm^−2^ s^−1^), and the pupil diameter of the left eye was recorded using an infra-red eye-tracking system. The order of light exposure (469 nm and 631 nm) was randomized and counterbalanced within each group of participants. All subjects completed the chromatic pupillometry test during the daytime between 8:30 am and 5:00 pm. To assess the amplitude of pupillary constriction, the pupil diameter was expressed as a percentage of the dark pupil prior to light exposure. The median constriction response was then determined in 0.5 log unit bins from 7 to 14 log photons cm^−2^ s^−1^, which allowed for construction of dose-response curves to 469-nm light and 631-nm light in each subject.

### Pupillary constriction outcome measures

To examine potential age-dependent differences in spectral responses of the pupillary light reflex, we determined (1) the threshold irradiance for a 10% pupillary constriction response, (2) the effective dose (ED) required for a half-maximal pupillary constriction response (ED_50_) based on the fitted dose-response function, and (3) the slope parameter of the dose-response curve. Below we summarize how these measures were derived from each subject’s pupil diameter data:

#### Threshold irradiance for pupillary constriction

The threshold for the pupillary light response was defined as the photon density required to elicit a 10% constriction response, i.e., when pupil diameter reached 90% of the dark pupil size. In our experience, pupil diameter rarely fluctuates by more than this amount when studied either in darkness or in the presence of continuous light. Hence, a response that exceeds 10% can be attributed to the effect of the light stimulus on pupil size. This measure does not reflect the absolute sensitivity threshold for the pupillary light response, but rather the amount of light required to elicit a small, standardized constriction response.

#### Determination of the ED_50_ and slope parameters

In each subject, dose-response curves for pupillary constriction to 469-nm light and 631-nm light were fitted with a sigmoidal logistic regression model using the following equation:


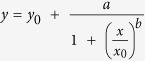


In this equation, *y*_*o*_ is 100% (the dark pupil size), *a* is the difference between 100% and the minimum response (expressed as percentage of dark pupil diameter), *x*_*o*_ is the log photon density that elicits a half-maximal response (ED_50_), and *b* is the slope parameter of the fitted dose-response function. Dose-response curve parameters were estimated based on the minimum sum of squares of the residuals using Sigmaplot 12.0 software (Systat Software, Inc., San Jose, CA).

### Data analysis and statistics

Pupillary constriction outcome measures (threshold irradiance, ED_50_, and slope parameter) were analyzed using ANOVA, with age as a between-subject factor (young, older) and wavelength as a within-subject factor (469 nm, 631 nm). A mixed ANOVA was also used to examine the interaction between cataract severity (none, mild, severe) and wavelength on pupillary constriction outcomes in older adults. For each ANOVA, when the omnibus test reached statistical significance, the Holm-Sidak method was used to perform pairwise multiple comparisons. The threshold for significance for all statistical tests was set at α = 0.05. Data processing and statistical analyses were performed using Matlab (Release 2013a, The MathWorks, Inc., Natick, Massachusetts, United States), Sigmaplot 12.0, and SPSS Version 22.0 software (IBM Corp., Armonk, NY).

## Results

### Subject characteristics

Of the 68 young normal subjects (aged 21–30 years) who completed the chromatic pupillometry test, 6 individuals were excluded from the analysis because their dark pupil diameter differed between light exposure trials by more than 10%. This criterion ensured that pupillary constriction responses could be compared reliably between blue and red light stimuli in each participant. Technical problems resulted in data loss in an additional 2 subjects. Therefore, 60 young normal adults were included in the analysis. Of the 300 older subjects who were studied in the polyclinic, 68 individuals were excluded due to diagnosis of an ophthalmic condition other than cataract, 43 individuals were excluded based on having an unstable baseline pupil diameter between light exposure trials, and 10 individuals were excluded due to technical problems during the pupillometry recording. Of the remaining 179 older subjects who were included in the analysis, 54 individuals were classified as normal with no cataract (LOCS grade 1), 107 individuals had mild cataract (LOCS grade 2), and 18 individuals had severe cataract (LOCS grade 3). Demographic characteristics of subjects are presented in Table 1.

### Spectral responses of the pupil in young versus older adults without cataract

**Figure 1:**
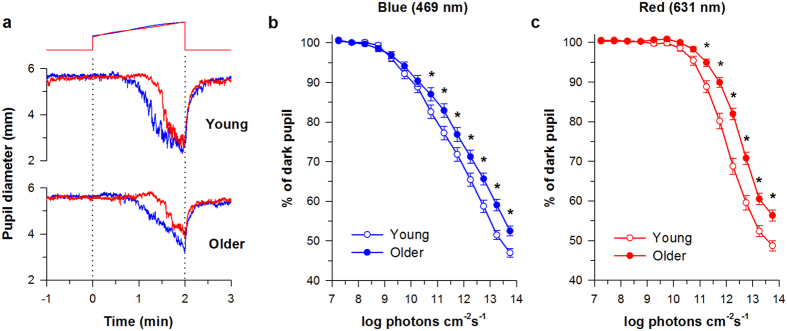
Pupillary light responses in aging. (**a**) Pupil diameter is shown in a representative young adult and in an older adult exposed to a 4-min light exposure sequence. Each trial consisted of 1 min of darkness, 2 min of monocular exposure to a gradually increasing blue light (469 nm) or red light (631 nm) stimulus, and 1 min of darkness after light offset. The timing of the ramp-up light stimulus is shown at the top of the plot. Dose-response curves for pupillary constriction are shown for young normal subjects (*n* = 60, aged 21–30 years) and older subjects without cataract (*n* = 54, aged 50–74 years) exposed to either (**b**) blue light or (**c**) red light. In each panel, the mean ± SEM is shown and asterisks indicate significant differences between age groups.

In both young and older adults, pupil diameter decreased gradually as the irradiance of the ramp-up light stimulus was increased over time (Fig. 1a). Based on dose-response curves, pupillary constriction was reduced in older individuals for blue and red light stimuli (Fig. 1b,c). There was a significant interaction between age and photon density, in which the age-related reduction in pupillary responses to blue light occurred at higher irradiances (*F*_13,1454_ = 6.31, *p* < 0.001 for interaction; *t* > 2.51 and *p* ≤ 0.012 for all pairwise comparisons above 10.5 log photons cm^−2^ s^−1^). Similar results were obtained for the red light stimulus, in which older subjects exhibited a reduction in pupillary responses at higher irradiances (*F*_13,1453_ = 15.73, *p* < 0.001 for interaction; *t* > 3.93 and *p* < 0.001 for all pairwise comparisons above 11.0 log photons cm^−2^ s^−1^).

Because the baseline pupil diameter was significantly smaller in older versus younger adults (Mean ± SD: young, 6.58 ± 0.88 mm; older, 5.30 ± 0.79 mm; Student’s *t*-test, *t* = −8.12, *p* < 0.001), we considered the possibility that reduced pupillary responses in older subjects could be explained by decreased retinal illumination caused by age-related miosis. We therefore performed a secondary analysis that included 20 young subjects and 20 older subjects with normal ocular health who were matched by their pupil size in darkness (±0.25 mm) (See Supplementary Fig. S1). Consistent with our previous analysis, pupillary constriction was reduced at higher photon densities in older subjects, even when the dark pupil size was similar to young adults prior to light exposure (*F*_13,493_ = 2.80 for the interaction; *p* < 0.001; *t* > 2.37 and *p* < 0.02 for pairwise comparisons between 12.0 and 13.5 log photons cm^−2^ s^−1^ for 469-nm light and *F*_13,494_ = 4.27 for the interaction; *p* < 0.001; *t* > 2.70 and *p* < 0.005 for pairwise comparisons between 12.0 and 14.0 log photons cm^−2^ s^−1^ for 631-nm light).

Contrary to our prediction that aging would result in a greater reduction in pupillary responses to blue light relative to red light, there was no significant interaction between age and wavelength on the threshold photon density required for a 10% constriction response (*F*_1,112_ = 0.24, *p = *0.62), the ED_50_ of the best-fit dose-response function (*F*_1,112_ = 0.86, *p *= 0.36), or the slope parameter of the fitted dose-response curve (*F*_1,112_ = 0.25, *p *= 0.62). We therefore turned our attention to main effects of age and wavelength on pupillary light responses (Table 2). Although there was a significant main effect of age on the threshold irradiance required to elicit a 10% constriction response (*F*_1,112_ = 3.98, *p *= 0.049), pairwise differences for each wavelength of light failed to reach statistical significance after correcting for multiple comparisons (469 nm, *t* = 1.12, *p *= 0.26; 631 nm, *t* = 1.79, *p *= 0.074) (Table 2). A main effect of age was also observed on the ED_50_ (*F*_1,112_ = 8.81, *p* < 0.01), with higher values observed in older participants, indicating reduced sensitivity to light (i.e., more photons were required for an equivalent pupillary constriction response in older subjects). Multiple comparison testing revealed a significantly greater ED_50_ value in older subjects exposed to the red light stimulus (*t* = 2.90, *p* < 0.01), whereas the difference in ED_50_ between age groups did not reach statistical significance for the blue light stimulus (*t* = 1.75, *p* = 0.081). There was no significant main effect of age on the slope parameter of the fitted dose-response curve (*F*_1,112_ = 0.36, *p* = 0.55).

For all pupillary response measures in young and older adults, there was a significant main effect of wavelength (Table 2), in which the blue light stimulus was associated with a lower threshold irradiance for 10% constriction (*F*_1,112_ = 66.2, *p* < 0.001), a lower ED_50_ value (*F*_1,112_ = 42.4, *p* < 0.001), and a shallower slope of the dose-response curve (*F*_1,112_ = 138.0, *p* < 0.001) relative to exposure to the red light stimulus. Pairwise comparisons revealed that these wavelength-dependent differences in pupillary light responses were significant in both young and older subjects (*t* > 4.06 and *p* < 0.001 for all pairwise comparisons).

### Spectral responses in eyes with cataract

**Figure 2:**
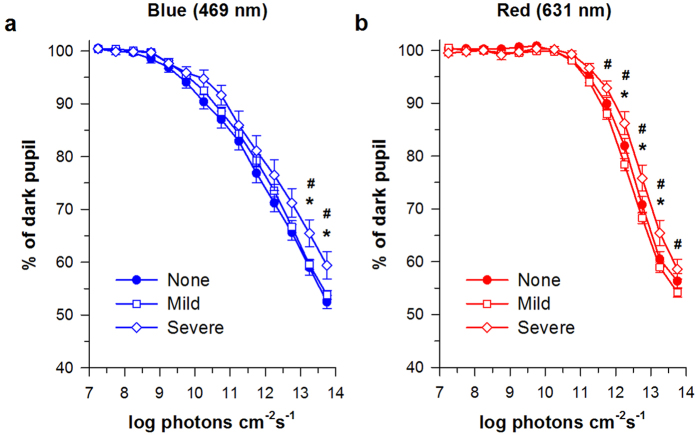
Pupillary light responses in patients with cataract. Dose-response curves for pupillary constriction are shown for older subjects (≥50 years) without cataract (*n* = 54), with mild cataract (*n* = 107), and with severe cataract (*n* = 18) exposed to a gradually increasing (**a**) blue light (469 nm) or (**b**) red light (631 nm) stimulus. In each panel, the mean ± SEM is shown. Asterisks indicate significant differences between patients without cataract versus those with severe cataract, and hashes (#) indicate significant differences between patients with mild cataract versus those with severe cataract.

Next, we examined the effect of cataract on pupillary constriction responses across different photon densities (Fig. 2). There was a significant interaction between cataract status and photon density on pupillary responses to blue light (Fig. 2a; *F*_26,2288_ = 1.52, *p *= 0.044). Multiple comparison testing revealed significant differences in pupillary constriction at higher irradiances between older individuals without cataract versus those with severe cataract (*t* > 2.74 and *p* < 0.05 for pairwise comparisons above 13.0 log photons cm^−2^ s^−1^), and between patients with mild cataract versus those with severe cataract (*t* > 2.53 and *p* < 0.05 for pairwise comparisons above 13.0 log photons cm^−2^ s^−1^). Similarly, there was a significant interaction between cataract status and photon density on pupillary responses to red light (Fig. 2b; *F*_26,2288_ = 2.82, *p < *0.001), with differences in pupillary constriction observed at higher irradiances between subjects without cataract versus those with severe cataract (*t* > 2.31 and *p* < 0.05 for pairwise comparisons from 12.0–13.5 log photons cm^−2^ s^−1^), and between patients with mild cataract versus those with severe cataract (*t* > 2.51 and *p* < 0.05 for pairwise comparisons above 11.5 log photons cm^−2^ s^−1^).

Next, we examined potential interactive effects of cataract grade (none, mild, severe) and wavelength (469 nm and 631 nm) on pupillary constriction responses in older individuals (Table 2). There was no significant interaction between cataract grade and wavelength on the threshold photon density required for a 10% constriction response (*F*_2,176_ = 0.59, *p *= 0.56), the ED_50_ of the best-fit dose-response function (*F*_2,176_ = 1.47, *p = *0.23), or the slope parameter of the fitted dose-response curve (*F*_2,176_ = 0.25, *p *= 0.78). Additionally, there was no significant main effect of cataract grade on the threshold irradiance for pupillary constriction (*F*_2,176_ = 0.22, *p *= 0.80), the ED_50_ (*F*_2,176_ = 1.69, *p *= 0.19), or the slope parameter (*F*_2,176_ = 0.50, *p *= 0.61). For each of the pupillary response measures examined, a significant main effect of wavelength was observed in which the blue light stimulus was associated with a lower threshold irradiance (*F*_1,276_ = 49.1, *p* < 0.001), a lower ED_50_ value (*F*_1,276_ = 46.9, *p* < 0.001), and a shallower slope of the dose-response curve (*F*_1,276_ = 127.5, *p* < 0.001) relative to the red light stimulus. These wavelength-dependent differences in pupillary light responses were observed in patients without cataract, as well as in patients with mild or severe cataract (*t* > 2.0 and *p* ≤ 0.037 for all pairwise comparisons).

## Discussion

Despite the marked reduction in retinal exposure to blue light that occurs in aging, we found that pupillary responses in older individuals were not impaired by a greater amount during exposure to blue light relative to red light. Pupillary constriction in older adults was reduced at higher irradiances compared with young adults, but this result did not depend on the wavelength of light. Hence, spectral responses (i.e., wavelength-dependent responses) for pupillary constriction were similar in young and older adults. These findings raise the possibility that age-dependent compensatory mechanisms preserve pupillary responses to short-wavelength light.

In this study, pupillary constriction responses to blue light were not selectively reduced in aging or in the presence of cataract, even though it is well established that more blue light is absorbed by the lens in older individuals^6^,^13^,^14^,^15^,^16^. Our findings are consistent with a recent study in which the magnitude of sustained pupillary constriction responses to blue light and green light stimuli (480 nm and 550 nm; 12.8, 13.5, or 14.0 log photons cm^−2^ s^−1^) did not differ between young and older subjects (18–30 years versus ≥55 years)^17^. Similarly, another study found that the post-illumination pupillary response (PIPR), which is thought to reflect sustained activation of melanopsin after light offset, was preserved and perhaps enhanced with increasing age across 4 decades of life (26 to 68 years)^18^. Together with our findings, these results suggest that the amplitude of melanopsin-dependent (λ_max_ = ~480 nm) pupillary responses shows little or no change across adulthood.

Only a few studies have examined whether circadian and melatonin suppression responses, which are also mediated by ipRGCs, exhibit altered spectral responses in aging. Previously, it was reported that the melatonin suppression response to blue light (456 nm) was reduced in older women compared with young women (Mean ± SD = 24 ± 3 years versus 57 ± 5 years)^8^. A similar reduction in melatonin suppression was observed in response to green light (548 nm) but did not reach statistical significance, perhaps due to high variability in the melatonin assay and the small sample size. In a subsequent study, circadian phase-advance responses to 456-nm light and 548-nm light did not differ significantly between young and older men (Mean ± SD = 23 ± 3 years versus 66 ± 5 years)^19^. Additionally, a recent study showed that young and older subjects (24–27 years versus 55–63 years) exhibited similar melatonin suppression responses to short-wavelength light ( < 500 nm, 13.5 log photons cm^−2^ s^−1^), despite a 42.3% reduction in lens transmission for 480-nm light in older subjects^20^. These studies suggest that lens yellowing in aging might have little effect on the magnitude of non-visual light responses. Hence, other factors are likely to be more important in age-dependent modulation of non-visual light responses, such as changes in autonomic nervous system function and loss of retinal ganglion cells^21^,^22^,^23^,^24^,^25^.

The preservation of pupillary responses to blue light in aging might seem paradoxical because prior work has shown that non-visual light responses are reduced when blue light is experimentally blocked from reaching the retina. For example, selectively blocking exposure to short-wavelength light (e.g., using specialized goggles) has been shown to prevent the suppressive effects of moderately-bright white light on melatonin production^26^,^27^,^28^,^29^. Analogous findings have been reported for the pupillary light reflex, in which filtering of short-wavelength light (460 nm to 480 nm) reduced sustained pupillary constriction responses to a bright blue light stimulus (470 nm, 100 cd m^−2^)^30^. In contrast to studies that have examined the immediate effects of a blue-blocking intervention (i.e., over minutes or hours), a recent study showed that blocking short-wavelength light ( < 550 nm) continuously for 2 weeks altered the sensitivity of melatonin suppression^31^. This was determined by having young subjects (Mean ± SD = 23.5 ± 4.6 years) wear a soft orange contact lens, which reduced exposure to 480-nm light by 50%. Exposure to moderately-bright white light (600 lux) failed to elicit melatonin suppression immediately after the contact lens was first worn, but the same light stimulus induced melatonin suppression after the contact lens was worn for 2 weeks. These results suggest that compensatory or adaptive mechanisms might boost the sensitivity of non-visual responses when short-wavelength light is blocked continuously for long time intervals. It remains to be determined whether this adaptive mechanism in young adults can account for compensatory changes in non-visual light responses with aging. The underlying mechanisms might be different because older individuals are exposed to gradually-reduced light transmission over the course of decades as opposed to weeks, with changes in retinal function occurring over many years.

Increased scattering of short-wavelength light with aging may help to preserve pupillary responses to blue light, as this would lead to illumination of a greater retinal area compared with younger individuals^32^. In the present study, however, age-dependent changes in light scatter are unlikely to explain our results because the eye was exposed to full-field retinal stimulation. Previous studies have shown that the effects of a light stimulus on circadian resetting and melatonin suppression are greater if preceded by dim light, and attenuated if preceded by brighter light^33^,^34^,^35^,^36^. The mechanisms underlying these changes in sensitivity remain unclear and have not been tested specifically for short-wavelength light, but it is plausible that a similar process could occur in aging to preserve non-visual responses when retinal exposure to blue light is diminished. For example, melanopsin-dependent ipRGC responses exhibit light adaptation and dark adaptation in mice^37^, and prolonged exposure to light or darkness has been shown to modulate melanopsin protein levels in neonatal rats^38^. Additionally, it has been reported that in patients with unilateral non-arteritic ischemic optic neuropathy, the PIPR is reduced in the affected eye but enhanced in the non-affected eye, suggesting a possible upregulation of melanopsin-dependent responses^39^. Based on our results for sustained pupillary constriction, as well as experiments performed by others on the PIPR and melatonin suppression^18^,^20^, the sensitivity of melanopsin-dependent responses might increase gradually with aging to compensate for the gradual reduction in blue light reaching the ipRGCs. Such adaptive changes could potentially occur in the retina itself, or in downstream targets of ipRGCs. Additional studies are needed to test whether such changes occur, and to delineate the mechanisms involved in preserving spectral responses with aging.

One of the limitations of our study is that lens transmission data were not collected. However, it is well established by measurements in donor eyes and live eyes that there is a marked increase in absorption of short-wavelength light in the aging lens^13^,^14^,^15^,^16^. Based on prior studies of lens transmission in similar age groups as those studied here^18^,^20^, we would expect at least a 40% reduction in the amount of 480-nm light transmitted to the retina in our older subjects without cataract, and a further reduction in blue light reaching the ipRGCs in patients with mild or severe cataract. Another limitation of our study is that young subjects did not undergo the comprehensive ophthalmic examination conducted in older participants. Therefore, we cannot exclude the possibility that some younger subjects had a health condition that affected pupil size. Additionally, our subjects in the older group were selected from a convenience sample that took part in a larger study that aimed to evaluate the incidence of ocular abnormalities in patients seeking outpatient care for non-ocular health issues^10^. Therefore, the number of individuals was not balanced across groups for cataract grade, and young adults were recruited separately from the general population. Previous research has also shown that light exposure patterns differ between healthy young and older adults^40^. Because we did not measure the light levels that participants were exposed to prior to the chromatic pupillometry test, we cannot exclude the possibility that differences in light history affected the dose-response for pupillary constriction.

Our light exposure protocol enabled us to construct dose-response curves to light over a short time interval, but the period of darkness before each light exposure trial was likely too short for complete dark adaptation of rod/cone photoreceptors^41^. This may have resulted in reduced sensitivity to light and masking of age-dependent differences in pupillary responses at low irradiances. Both visual photoreceptors and melanopsin contribute to sustained pupillary light responses^42^,^43^,^44^, but these photoreceptor types play different roles: rod-cone input is required for normal pupillary constriction at lower irradiances and in response to long-wavelength light, whereas melanopsin is required for normal pupillary responses to high-irradiance short-wavelength light^45^,^46^. We found that older individuals exhibited reduced responses to red light outside the range of sensitivity for melanopsin, as well as reduced responses to high-irradiance blue light^45^,^47^,^48^. Together, these findings are consistent with reduced input from both visual photoreceptors and melanopsin to ipRGC responses and/or reduced function of downstream elements in the pupillary light reflex pathway.

The ramp-up light exposure protocol that we used revealed age-dependent differences in pupillary responses at higher irradiances (i.e., > 10.5 log photons cm^−2^ s^−1^). During the early part of the light stimulus (i.e., at the lowest irradiances tested, < 9.0 log photons cm^−2^ s^−1^), there was no difference between age groups because there was no detectable change in pupil size (Fig. 1). The amplitude of pupillary constriction was similar between young and older adults during the initial light response (from about 9.0 to 10.5 log photons cm^−2^ s^−1^), when the pupil was 90–95% of the dark pupil size. Within this narrow range of irradiances, age-dependent differences may have been too small to detect, or rod/cone input was sufficient to drive normal pupillary constriction. Pupillary responses diverged at higher irradiances, with older individuals exhibiting reduced pupillary constriction across most of the dynamic range of the sustained pupillary light reflex (i.e., corresponding to the log-linear portion of the dose-response curve). A further attenuation of pupillary responses to blue and red light stimuli was observed in patients with severe cataract, which is consistent with reduced light activation of rod/cone photoreceptors and melanopsin.

In our experiments, the direct pupillary light reflex was measured, i.e. pupil diameter was measured from the same eye that was exposed to light. An alternative approach would be to pharmacologically dilate the pupil exposed to light, and then measure the consensual light reflex from the other eye. This would have the advantage of minimizing age-dependent differences in baseline pupil size that can influence the magnitude of pupillary light responses. However, previous studies have shown that pupil size in older subjects is smaller compared with younger subjects even when a mydriatic agent is used to dilate the pupil^49^. Our sample was sufficiently large that we were able to perform a sub-analysis with young and older subjects who were matched by their dark pupil size. Under the conditions used in our study, with a pupil that was free to constrict as in the real world, we found no functional consequence of aging on spectral responses of the pupil. We cannot exclude the possibility that there was a small age-dependent shift in the peak spectral sensitivity of pupillary light responses because we only compared dose-response curves for blue and red light stimuli. However, if such a shift occurred with aging, it was not great enough to selectively influence pupillary responses to blue light.

Finally, it should be highlighted that our results for the pupillary light reflex might not reflect what occurs for other non-visual light responses. Some melanopsin-containing cells send branched projections to the pretectal area and the master circadian clock in the suprachiasmatic nucleus^50^, but there are also distinct subpopulations of ipRGCs that project to these brain sites^51^. Although these pathways could be differentially affected in aging, pupil size measured during exposure to light has been shown to correlate with the magnitude of melatonin suppression in humans^52^. Additionally, the amplitude of the PIPR after exposure to blue light has been shown to correlate with the midpoint of nocturnal sleep under free-living conditions, which is often used as an estimate of circadian timing^53^. These findings suggest that pupillary responses might serve as a surrogate marker for other non-visual light responses, even though pupillary constriction, melatonin suppression, and circadian responses exhibit different threshold sensitivities^54^.

Aging is associated with reduced sensitivity of the circadian system to light, altered timing of circadian rhythms relative to nocturnal sleep, and increased sleep disturbances^55^,^56^,^57^. Based on the premise that lens yellowing might result in decreased activation of ipRGCs, it has been proposed that insufficient retinal exposure to blue light contributes to sleep problems in aging^6^. An inverse relationship has been reported between lens transmission determined by autofluorescence and sleep disturbances^9^, but there are many age-related factors (ocular and nonocular) that can potentially influence the quality of sleep in older individuals. Our findings for the pupillary light reflex, and recent studies on the PIPR and melatonin suppression^18^,^20^,^31^, challenge the widely-held assumption that yellowing of the lens has important functional consequences for non-visual responses to blue light. These results have potential implications for light therapy-based treatment of circadian sleep disturbances and cognitive dysfunction in older populations^58^,^59^. While it might be expected that older patients would need brighter light or more blue-enriched light relative to younger individuals to reset their circadian clock and to consolidate sleep, it has yet to be clearly demonstrated that circadian and other behavioral responses to blue light are selectively reduced in aging. Therefore, additional studies are needed to determine whether light therapy can be optimized to minimize patient burden and improve sleep and well-being in older populations.

Our findings may help to inform the ongoing debate on whether blue-blocking intraocular lenses (IOLs) have a negative impact on non-visual light responses. Cataract is treated by removing the opacified lens and, in most cases, replacing it with an artificial IOL. Blue-blocking IOLs mimic the natural yellowing of the lens that occurs in aging, which some researchers have argued could reduce the risk of blue light-induced retinal damage and protect against age-related macular degeneration^60^. Critics have raised the issue that filtering out too much blue light could result in circadian sleep disturbances due to reduced activation of ipRGCs^3^. In a clinical trial comparing the impact of blue-blocking IOLs and neutral IOLs, there were no between-group differences in the 24-h profile of melatonin or actigraphy-estimated sleep measures^61^. Following surgery, there was a small increase in the PIPR to blue light, which is consistent with our observation that pupillary responses were reduced by a small amount at high irradiances in patients with severe cataract. However, the PIPR did not differ between patients with blue-blocking IOLs versus neutral IOLs. Other studies have found that short-term improvements in sleep quality and depression scores following lens replacement are equivalent for IOLs that filter blue light versus those that only block UV light^62^,^63^,^64^. These findings suggest that reduced transmission of blue light with IOLs might have limited clinical impact on non-visual light responses, similar to our findings for the pupillary light reflex in nonpathalogical aging.

In conclusion, our study demonstrates that aging and cataract do not selectively impair pupillary responses to blue light. Hence, melanopsin-dependent pupillary constriction is largely preserved in aging. If our findings extend to circadian responses, which are also mediated by ipRGCs, yellowing of the lens might contribute little to age-related sleep problems. Presumably, compensatory mechanisms occur in aging that preserve spectral responses of the pupillary light reflex. This might be analogous to compensatory mechanisms that preserve hue perception across the lifespan^65^,^66^, raising the possibility that both visual and non-visual photoreceptor pathways adapt to ocular changes in lens transmission to ensure relative constancy of light responses.

## Additional Information

**How to cite this article:** Rukmini, A. V. *et al*. Pupillary responses to short-wavelength light are preserved in aging. *Sci. Rep.* **7**, 43832; doi: 10.1038/srep43832 (2017).

**Publisher's note:** Springer Nature remains neutral with regard to jurisdictional claims in published maps and institutional affiliations.

## Supplementary Material

Supplementary Figure S1

## Figures and Tables

**Table 1:** Demographic information for study participants.

Subject group	*n*	Number of males (%)	Number of Chinese subjects (%)	Age in years mean ± SD (Range)
Young	60	21 (35.0)	55 (91.7)	25.3 ± 2.4 (21–30)
Older, no cataract	54	19 (35.2)	49 (90.7)	56.4 ± 5.1 (50–74)
Older, mild cataract	107	36 (33.6)	102 (95.3)	61.5 ± 5.9 (50–78)
Older, severe cataract	18	7 (38.9)	18 (100)	63.9 ± 6.9 (54–81)

**Table 2:** Pupillary light responses in young versus older subjects.

	Young (mean ± SD)	Older No cataract (mean ± SD)	Older Mild cataract (mean ± SD)	Older Severe cataract (mean ± SD)
Threshold for 10% constriction				
Blue light (log photons cm^−2^ s^−1^)	9.72 ± 1.07	9.97 ± 1.27	9.91 ± 1.39	10.25 ± 1.57
Red light (log photons cm^−2^ s^−1^)	10.86 ± 1.05	11.26 ± 1.28	11.14 ± 1.16	11.06 ± 1.44
ED_50_ for half-maximal response				
Blue light (log photons cm^−2^ s^−1^)	11.57 ± 0.87	11.81 ± 0.93	11.97 ± 0.78	12.05 ± 0.85
Red light (log photons cm^−2^ s^−1^)	12.04 ± 0.56	12.43 ± 0.38*	12.37 ± 0.41	12.69 ± 0.36
Fitted slope parameter				
Blue light (unitless)	−15.29 ± 5.79	−16.47 ± 7.04	−17.06 ± 6.89	−18.16 ± 8.90
Red light (unitless)	−28.33 ± 9.34	−28.45 ± 9.71	−27.84 ± 8.20	−29.73 ± 8.74

The only significant difference in pupillary constriction between young and older subjects was for the ED_50_ of the dose-response curve to red light, highlighted by the asterisk (*). A significant main effect of wavelength (469-nm blue light versus 631-nm red light) on pupillary constriction was observed for all outcome measures in all groups. Additional statistical comparisons are described in the main text.
