# Supplementary material for: Pupillary responses to short-wavelength light are preserved in aging
Source: Sci Rep. 2017 Mar 7;7:43832. doi: 10.1038/srep43832 (PMC5339857; doi:10.1038/srep43832)
Supplement: Supplementary Figure S1 [file srep43832-s1.pdf]

Supplementary Information

Pupillary responses to short-wavelength light are preserved in aging

A.V. Rukmini, Dan Milea, Tin Aung, Joshua J. Gooley

Supplementary Fig. S1

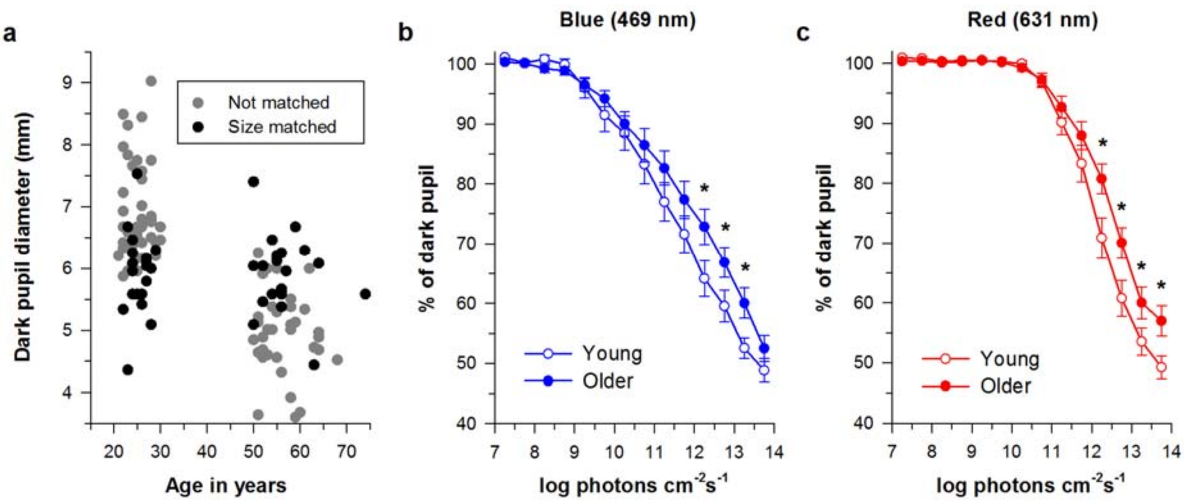

Fig. S1. Pupillary light responses in young and older subjects matched by their baseline dark pupil size. (**a**) The dark pupil diameter prior to light exposure is shown in young subjects (*n* = 60, aged 21-30 years) and older subjects without cataract (*n* = 54, aged 50-74 years). A subgroup of 20 subjects was selected randomly from each age group, matched by their baseline pupil size and order of light exposure (black circles). Dose-response curves for pupillary constriction are shown for these subjects during exposure to (**b**) blue light (469 nm) or (**c**) red light (631 nm). In each panel, the mean ± SEM is shown and asterisks indicate significant differences between age groups.
